# The NIST Cold Neutron Research Facility

**DOI:** 10.6028/jres.098.001

**Published:** 1993

**Authors:** H. J. Prask, J. M. Rowe, J. J. Rush, I. G. Schröder

**Affiliations:** National Institute of Standards and Technology, Gaithersburg, MD 20899

**Keywords:** cold neutrons, guide hall, neutron facilities, neutron guides, neutron instrumentation, neutron properties, neutrons, research reactors

## Abstract

The Cold Neutron Research Facility (CNRF) at the National Institute of Standards and Technology (NIST) Research Reactor (NBSR) is now coming on line, with the first seven experimental stations operational, and more stations scheduled to be installed during 1992. The present article provides an introduction to the facility, and to other articles in the current issue that give more details on some of the research opportunities that the facility will bring to NIST.

## 1. Introduction

Since the advent of nuclear reactors with reasonably high fluence rates (fluxes) in the 1950s, the use of the thermal neutrons produced by these sources to study a wide range of problems has grown rapidly. This growth has resulted from the properties of neutrons as probes, which include:

- absorption with subsequent radioactive decay (used as an analytical tool)
- high penetration as a result of the lack of an electrical charge
- scattering by nuclear interactions (which leads to a *non* monotonic variation of scattering with atomic number)
- special sensitivity to hydrogen and hydrogenous materials along with strong difference in scattering by hydrogen and deuterium
- scattering by magnetic interactions (as a result of the magnetic dipole of the neutron)
- simple interpretation of results (as a result of the weak interaction)
- the nondestructive nature of low energy neutrons
- the fact that neutrons with wavelengths comparable to interatomic spacings in condensed matter have energies comparable to the energies of atomic vibrations.

The potential of this research was realized early, and the Atomic Energy Commission built a succession of research reactors with increasing fluence rates throughout the 1950s and 1960s.

This potential was also perceived by three physicists at the National Bureau of Standards (NBS, now NIST)-R. S. Carter, H. Landon, and C. Muelhause—who proposed, designed, and built the 20 MW research reactor, NBSR, on the new NBS site in Gaithersburg. The original proposal was justified on the importance of the contributions that the NBSR would provide to the NBS mission in analytic chemistry, standards, solid state physics, and nuclear physics, and was intended to serve NBS and other government agencies in the Washington area. The reactor design emphasized versatility, incorporating a large number of experimental facilities with different capabilities, rather than concentrating on any one specific area of research. The success of this philosophy can be seen both in the increasing utilization of the reactor by NIST and other researchers, and in the changing emphasis in programs over the years. The design also included many advanced features that have since been incorporated into other designs, including a completely passive emergency cooling system, a novel split core fuel design (recently adopted into designs for the Advanced Neutron Source and the spallation source at Los Alamos National Laboratory), helium and carbon dioxide blankets around high flux regions to reduce radioactive emissions and the use of large radial penetrations into the high flux regions to maximize intensity for beam research.

**Fig. 1:**
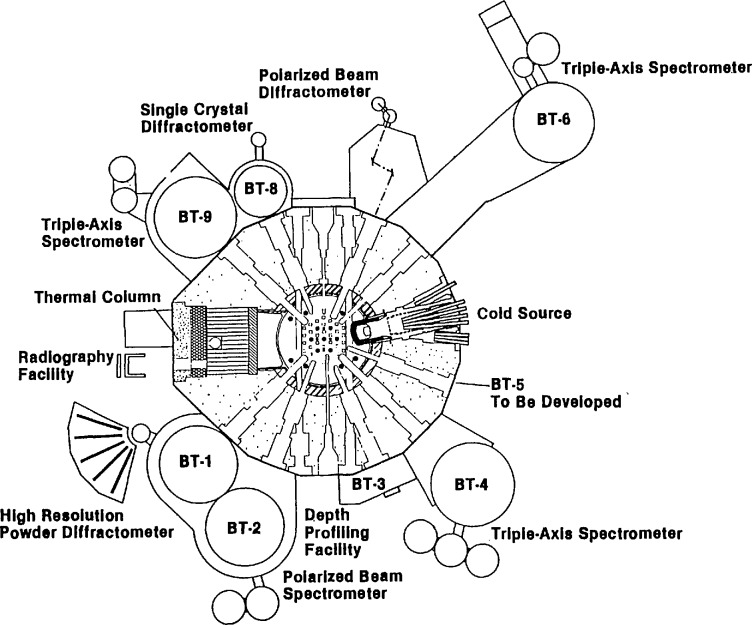
Principal experimental facilities in the reactor hall.

Since the reactor first achieved continuous operation in 1969, the use of the facilities has grown steadily in both the number of researchers served (over 450 research participants the last year before the CNRF was operational) and in the breadth of the community served (the 450 participants came from 15 NIST divisions or offices, 25 other government agencies, 50 universities and 25 industrial laboratories). The NBSR has gone from a local resource to a truly national facility, with researchers from all over the country. A diagram of the current layout of facilities around the reactor is shown in Fig. 1.

**Fig. 2:**
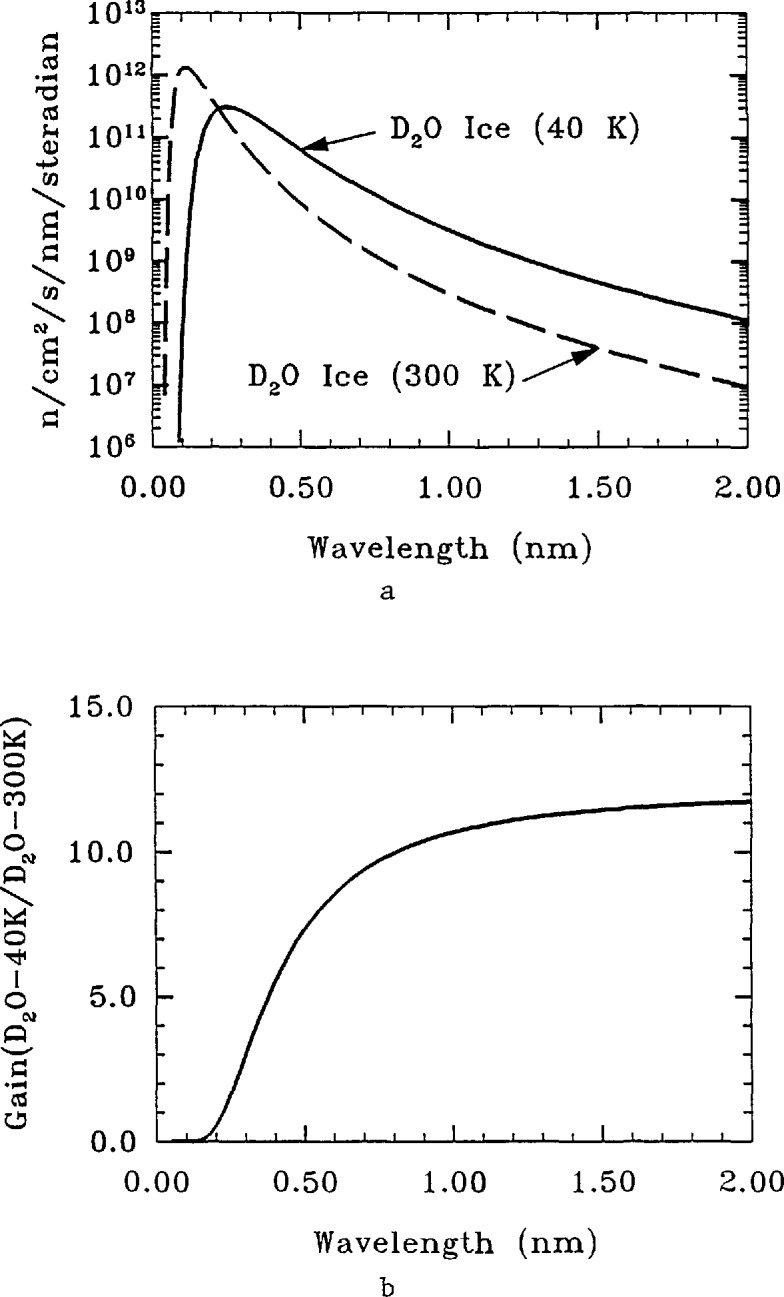
(a) Neutron fluxes for the cold moderator cavity filled with liquid D_2_O and D_2_O ice. (b) Flux gain, D_2_O(ice)/D_2_O(liquid), from the data of (a). The moderator temperature of 40 K produces a Maxwellian distribution with an “effective” temperature of 60 K.

One of the features incorporated into the design was the large penetration for a “cold neutron source” shown in Fig. 1. From the beginning, the designers intended that this penetration would one day be used to provide an intense source of neutrons with lower energies than those typical of other ports by installation of a cryogenically cooled block of neutron moderator. A moderator for neutrons is a material which scatters the neutrons produced by fission so that their energy is reduced from the MeV energies typical of nuclear reactions down to the meV energies typical of atomic motions in thermal equilibrium at approximately room temperature. The energy spectrum produced by such moderators is a Maxwellian distribution with a characteristic temperature close to that of the moderator itself—hence the name thermal neutrons. An example of such a spectrum is shown in Fig. 2, with temperature chosen to represent the spectrum seen by the thermal beam tubes at the NBSR. Cold neutrons are defined as those with energies less than 5 meV, corresponding to 60 K and velocities less than 1000 m/s. However, this cold neutron port was not utilized at the beginning, and the development concentrated on the thermal neutron beams and facilities. In 1985, the development of the cryogenic port began in earnest with funding of a cold neutron initiative (made possible by preliminary funding from the NBS director in 1984). As discussed below, events have shown that the large area allowed for a cold source in the NBSR design and the very wide range of angular access (over 30°) was a key to providing a unique opportunity to create the only fully internationally competitive cold neutron research capability in the United States.

The interest in applications of cold neutron techniques was stimulated by the success of the Western European research reactors, most notably the Institut Laue Langevin (ILL) in Grenoble, France. Among the many successes of these facilities was the demonstration of the power of a technique called Small Angle Neutron Scattering (SANS) as applied to a wide variety of problems in polymers, biology, metallurgy, and other areas in which structures with distance scales in the 1–100 nm range were critical. The United States was highly competitive in atomic and molecular scale measurements with thermal neutrons, but was far behind in utilization of longer wavelength neutrons, and the SANS technique was the area addressed first. In 1979 one of the first competence proposals funded by the NBS director was to develop a SANS spectrometer and research program at the NBSR, which led to the 8 m SANS on a beam from the cold source port (but without a cold source installed). This facility was a success, and the use of the instrument for a wide variety of studies grew rapidly, with users from many parts of NBS and outside organizations. It was clear that the productivity and capability of this instrument could be dramatically increased by completion of the cold source itself, since the instrument used neutrons with velocities below 1000 m/s, and was therefore working with the tail of the 350 K Maxwellian spectrum characteristic of the NBSR moderator. At the same time, it was evident that many other measurements could be greatly improved by the use of cold neutrons, if the cold source were developed and space were available to develop the proper instruments.

Another of the developments that had been demonstrated at the ILL was the use of long neutron “guides” to conduct intense beams of neutrons 10’s of meters away from the reactor into large experimental halls (generally called guide halls). These guides are based on the total internal reflection of neutrons below a critical angle that is a function of the reflecting material and neutron energy. This reflection is exactly analogous to optical reflection, and the critical angle depends on the deBroglie wavelength of the neutron, which is inversely proportional to neutron velocity and equal to 0.4 nm at 1000 m/s. Thus, guides work best at long wavelengths and therefore for cold neutrons. The guides themselves consist of pipes which are made of optically flat glass coated with materials of high scattering power (generally an isotope of nickel) and which are evacuated in order to reduce losses due to air scattering. Using these devices, it is possible to transport intense beams of cold neutrons out of the reactor building with losses of less than 1%/m, thus opening up the possibility of creating many new types of measurement capability in a large new experimental hall.

Based on these considerations, NBS proposed a cold neutron facility for the FY1985 budget, and it was included in the budget submission to the Department of Commerce and OMB for that year. However, many proposals for new facilities were submitted at that time from different agencies, so the Office of Science and Technology Policy requested a national study to set priorities for these facilities. As a result, the National Academy of Sciences commissioned a study by a panel of the National Research Council on “Major Facilities for Materials Sciences and Related Disciplines” chaired by Professor F. Seitz and Dr. D. Eastman to set national priorities. At the same time, a smaller initiative to develop the cold neutron source at the NBSR and to conduct research using two prototype instruments was funded in the FY1985 budget at the $1.5M level. With this funding, work on the cold source accelerated, and by 1987, the present cold source was installed and operating with the two prototype instruments — an improved 8 m SANS spectrometer and a time-of-flight spectrometer for studying dynamical processes. At the same time, the full NBS proposal for a Cold Neutron Research Facility was presented to the Seitz-Eastman committee.

In 1985, the committee reported its findings as a list of priorities divided into two different categories—new facilities, and new capabilities at existing facilities. In the last category, the proposals for cold neutron capabilities were given the highest priority for immediate funding. This priority was a recognition of the large contributions of cold neutron measurement to many areas of research, and of the fact that the United States was seriously behind in these techniques, at a time that Western Europe had invested over $150M in such facilities, and Japan was about to build a new reactor largely driven by cold neutron research needs.

In response, NBS and the Department of Commerce submitted the CNRF proposal for the FY1986 budget, and it was included in the President’s budget submission to Congress. Although the need for the facility was accepted and no technical faults were seen, this initiative was not funded for FY1986, and was resubmitted for FY1987. The original proposal was for a short project of 3 years, with a total construction budget of $25M. In FY1987, a proposal for the CNRF with the same budget, but stretched over 1987–1992 was approved by the Congress, and in the late fall of 1986, detailed design of the facility began. This approval gave NBS the mission of building a national facility for shared use by researchers from all over the nation that would make available the cold neutron measurement techniques that did not exist anywhere else in the United States. The CNRF that is now becoming operational is the result.

## 2. Cold Neutron Source

A research reactor operates by the fissioning of uranium, a nuclear reaction which produces neutrons of energies in the range of 1–2 MeV. However, the fission process itself requires neutrons of 8–9 orders of magnitude lower energies (25 meV) to proceed efficiently. For this reason, all thermal reactors require that the fuel be surrounded by “moderators”, which slow to thermal energies the neutrons produced by the fission process. These moderators are usually water—in the case of the NBSR, heavy water or D_2_O at approximately room temperature. Heavy water has a lower neutron capture cross-section than normal water (H_2_O) and so is often used in research reactors, where the goal is to produce the maximum number of neutrons possible for use in beams. At the same time, the moderator also provides neutrons which are the right energy for use in most neutron research, with a typical energy of 25 meV, corresponding to a velocity of 2200 m/s, and a deBroglie wavelength of 0.18 nm. The neutrons are used for experiments either by inserting samples into the reactor itself or by extracting beams through holes (beam tubes) which penetrate the shielding.

As shown in Fig. 2, even for the normal D_2_O moderator (*T*~40°C) there are some neutrons with energy below 5 meV (cold neutrons), but the number is small because they come from the tail of the spectrum. The number of these neutrons could obviously be increased by lowering the temperature of the moderator, and this is the basis for the idea of a cold neutron “source.” Of course, a cold neutron source does not generate cold neutrons—rather, it further moderates existing neutrons to lower effective temperatures. As a result, cold moderators or sources are never as efficient as might be expected on the naive idea of simply reducing the effective temperature. There are additional losses which reduce the gain by varying amounts, depending on the details of the reactor, moderator geometry, moderator properties, etc., and in any given case, different compromises must be made. Also, there are stringent safety requirements which impose additional constraints.

**Fig. 3:**
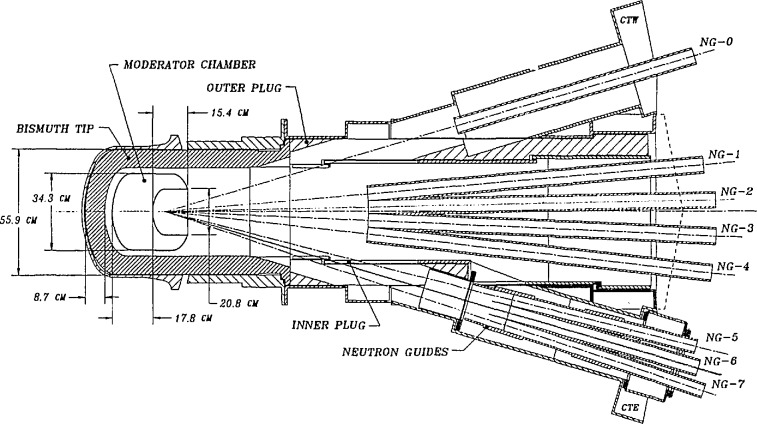
The D_2_O cold source at the NBSR.

A schematic diagram of the existing cold neutron source is shown in Fig. 3, with the neutron guides (see description below) installed. The main components are the lead/bismuth shield lining the beam port (required to reduce -γ-ray heating in the moderator), a cryostat containing the D_2_O ice (which is surrounded by an insulating vacuum and a helium blanket), and the cooling tubes (which carry helium gas at 30–40 K to cool the source). The other main component (not shown) is a helium gas refrigerator which supplies 1.0 KW of cooling at a helium mass flow rate of 28 g/s. The cryostat is fabricated of a magnesium alloy, in order to reduce the heat load on the system and increase the neutron efficiency. With the reactor operating at 20 MW, the helium gas enters the cryostat at 37 K and exits at 43 K, giving an average temperature in the ice (as a result of thermal conductivity) of approximately 45 K. This moderator was chosen for the first cold source in the NBSR on the basis of good neutron properties and safety considerations. The spectrum which this moderator produces is shown in Fig. 2, and the measured gain which it provides for cold neutrons is of the order of 10. (Gain is defined as the measured ratio of the number of cold neutrons produced with the moderator filled with D_2_O ice at low temperature to the number produced with the moderator filled with D_2_O at 300 K.) By direct experiment (as well as calculation), we have determined that this ratio is maximized by the addition of 7% H_2_O. The moderator is, in fact, operated this way. This moderator has been in service since 1987, and system reliability is now quite good (>98%availability). As a result of radiation damage of the solid ice, the moderator is warmed up to ∼80–100 K every 2 days to allow recombination of the constituents produced by radiolysis.

With this source in operation, we have turned our attention to a second generation source which will utilize liquid hydrogen. Such a source offers several advantages over the ice source, but also imposes several new constraints and safety issues. The primary gain comes from the fact that hydrogen remains liquid down to 14 K, and liquids, because of their diffusional motions, are—in general—better neutron moderators for low energies (the relatively low energy rotational levels of the hydrogen molecule are also extremely beneficial). The liquid source is also easier to operate, since the moderating material can be cooled outside the reactor, and flowed into the source. This simplifies the associated plumbing, since fewer lines must be maintained in high radiation fields. The main drawback comes from the necessity to handle relatively large volumes of hydrogen, with the potential safety problems that this implies. However, it should be noted that the entire inventory is the equivalent of less than one standard gas bottle.

**Fig. 4:**
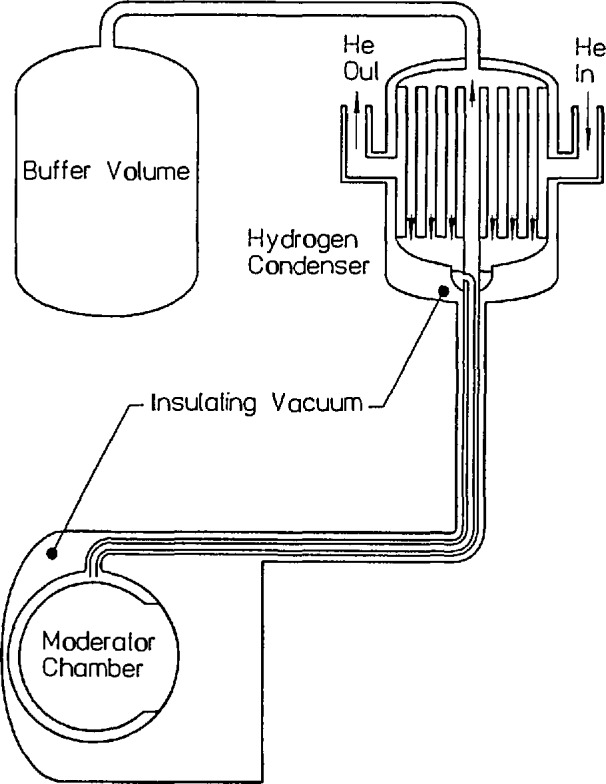
The H_2_ cold source to be installed at the NBSR.

A schematic drawing of the proposed hydrogen source is shown in Fig. 4, from which several features should be noted. First, as a result of the lower density of hydrogen, the lead/bismuth shield can be left out, with a resultant gain in intensity and simplicity. Second, the source itself is much thinner, as a result of the high scattering power of hydrogen. The moderator chamber is a 2 cm thick, spherical shell with an outside diameter of 32 cm, containing about 5 L of liquid hydrogen. A 20 cm diameter reentrant hole fully illuminates the guides with cold neutrons from the flux-trap in the interior of the sphere. Safety and simplicity are the dominant design considerations of the source, shown schematically in Fig. 4. A large buffer volume is open to the moderator chamber so that the entire liquid hydrogen inventory can vaporize and expand into the buffer without over-pressurizing the system. The cooling mechanism is a gravity fed flow of liquid into the moderator chamber, where evaporation removes the heat produced by the radiation. The hydrogen is in a cooled loop, reliquified outside the beam port, completing the naturally circulating thermosyphon requiring no pumps or moving parts. The entire hydrogen system is surrounded by an insulating vacuum, which is in turn surrounded by gaseous helium (not shown in Fig. 4) in order to prevent the entry of oxygen into any volume containing hydrogen. This is a central part of the safety philosophy—namely, to limit the oxygen available for combination with hydrogen. The liquefaction is done in the hydrogen condenser, with cooling provided by a 3.5 KW helium gas refrigerator. The first engineering tests of the proposed new source will take place this summer, and a full safety analysis is being prepared for submission to the Nuclear Regulatory Commission this year. Calculations of the performance of this source (performed both by analytical and Monte Carlo methods) indicate a further gain of cold neutrons available for experiments of at least a factor of two over the existing source, with somewhat larger gains at the lower energies.

## 3. The Neutron Guides

Because of the wave properties of the neutron, it is necessary to consider an index of refraction for different materials in which neutrons propagate, and to take account of differences in these indices as the neutrons cross surfaces between materials of different properties. As originally shown by Fermi (see following paper by N. F. Berk), the index of refraction, *n*, of a material is given by

$$n^{2}=1-\lambda^{2}\cdot N\cdot a_{c}/\pi$$ (1)

where λ is the neutron wavelength, *N* is the number of atoms per unit volume, and *a_c_*is the coherent neutron scattering length $\left(\text{scattering}\;\text{cross-section}=4\pi a_{c}^{2}\right)$. Just as is the case for visible light, the difference in indices of refraction of two media can lead to total reflection at a surface. For the case of neutrons, this leads to an expression for the critical angle *γ*_c_ in terms of the neutron wavelength and materials properties (assuming a vacuum-material interface) as

$$\gamma_{c}=\lambda \cdot {\left(N\cdot a_{c}/\pi \right)}^{1/2}$$ (2)

with *γ*_c_ in radians. In contrast to visible light, typical values in the neutron case for *n* and *γ*_c_ are 10^−5^ and 1°, respectively. In principle, the reflectivity is unity for angles less than the critical one, but in practice the reflectivity is less than this for various reasons, such as surface roughness and variations in coating thickness. This phenomenon is the basis for the manufacture of “neutron guides,” which are used in the CNRF.

The actual neutron guides are long tubes of rectangular cross section (either 120 × 50 or 150 × 60 mm^2^), made from boron containing glass, polished on the inside to an rms surface roughness of 2 nm and a flatness equivalent to 10^−4^ rad, coated with approximately 80 nm of ^58^Ni (this isotope of nickel has the best neutron properties of any material readily available), and evacuated most of their length. The guides are manufactured commercially in 1500 mm lengths (which are in turn fabricated from 500 mm length components), and then aligned to the requisite 10^−4^ rad over the entire length (up to 70 m) by optical techniques after being mounted on I-beam supports. When aligned, the 1500 mm lengths are joined together by a silicone based sealant (the gaps are less than 0.25 mm), sealed at the ends by windows of Al or Mg, and helium leak tested. They are then evacuated by turbo-molecular pumps, and surrounded by neutron and *γ*-ray shielding. These guides conduct the neutrons from the cold source described above (see Fig. 3) out through the reactor confinement building walls into the experimental hall described below. In order to maintain the integrity of the reactor building containment, a “shutter” is inserted at the wall which closes automatically by gravity whenever the reactor is shut down, providing a complete seal of the building. These shutters also serve to interrupt the neutron beams when desired, so that work can be performed in the experimental hall while the reactor is operating.

Four guides, NG-3, NG-5, NG-6, and NG-7, have now been installed, and their performance has been measured, showing that the losses (due primarily to reflectivity losses resulting from surface roughness) are less than 0.7%/m. Thus, beams can be transported over several tens of meters (the longest guide is 71 m), with low loss. As a result, many new experimental stations can be built, increasing the utilization of a unique facility. In addition, since guides transport cold neutrons preferentially, unwanted radiation is reduced in inverse proportion to the square of the distance from the source.

The actual number of neutrons transmitted by the guides depends on the solid angle accepted, which is determined by the critical angle for total reflection, as defined above. For ^58^Ni, *γ*_c_ = 0.02 λ, where λ is the neutron wavelength in nm, and *γ_c_*is the angle of reflection in radians. Since both senses of angle are transmitted, the total divergence accepted is 2*γ*_c_., in both the horizontal and vertical directions, giving a solid angle of $4\gamma_{c}^{2}$ sterad. While this is quite adequate for many experiments using cold neutrons, larger divergences would be useful in some cases, and for shorter wavelength (thermal) neutrons, the transmission is almost too small to be useful.

For several years, coatings with larger critical angles have been sought, and in fact so-called super-mirrors have been developed which consist of many layers of materials with different neutron properties, arranged in a particular sequence. These devices can double, triple, or even quadruple the critical angle, but until recently, the reflectivities were too low for use in guides, where many reflections take place before a neutron reaches the end of the guide (the average number of reflections is directly proportional to the critical angle, and inversely proportional to the guide dimensions perpendicular to the beam direction). Researchers at NIST, in collaboration with Oak Ridge and Brookhaven National Laboratories, and with two small industrial firms, have recently demonstrated that these devices can be made with the requisite reflectivity for use in guides, at least on the laboratory scale. We are planning to use these coatings for at least the tops and bottoms of the three remaining guides.

## 4. The Experimental Hall and Associated Facilities

The new experimental hall, often referred to as the guide hall, is a slab on grade structure 61 m long × 30 m wide × 13.7 m high built adjacent to the north wall of the original reactor building. The design floor loading is ∼96000 N/m^2^ (2000 lb/ft^2^) in order to support the massive instruments and shielding associated with neutron beams. The walls, roof supports, and rails for a 20 ton crane are supported on augured cast-in-place piles, which are independent of the floor structure for vibration isolation. In addition, reinforced concrete beams, independently supported on their own piles, are laid out along the individual guide tube directions, providing a stable, vibration-free platform that allows the guides to retain their precision alignment to 10^−4^ rad even when heavy loads are moved on the experimental floor.

**Fig. 5:**
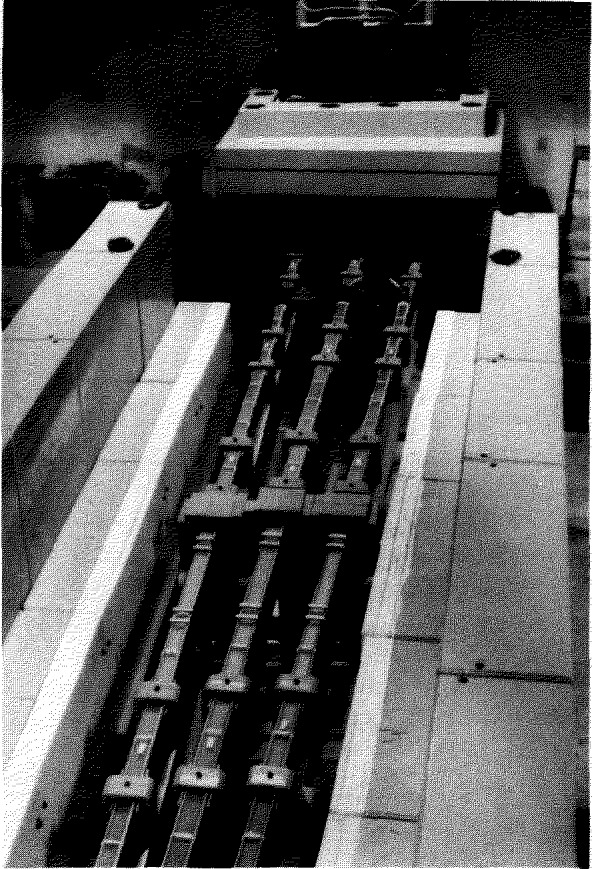
Neutron guides, NG-5, NG-6, and NG-7, in the NBSR reactor hall.

For radiation protection, the guides are surrounded along their entire length by specially constructed shielding consisting of welded steel tanks filled with either steel shot and paraffin or a paraffin-borax mixture. The shielding is divided into modular pieces with specially designed overlap so that at any point, the shielding can be removed in order to insert instruments as required. In Fig. 5, which is taken facing away from the reactor looking towards the wall, the first three guides (with top shielding removed) and the beam shutters can be seen.

In order to get the beams into the guide hall, it was first necessary to core-bore seven large diameter holes through the ~8 m thick reinforced concrete wall, after which a stainless steel liner was inserted and grouted into place. The guides were inserted into a 9 m long steel casing, aligned optically, and the entire casing-guide assembly was then aligned onto the guide direction. The ends of the casings are sealed to the reactor building walls, and the empty space between the casing and liners is filled with mineral oil, which is a good neutron shielding material. Outside the wall, the guides are individually assembled from 1.5 m sections supported on I-beams, aligned optically, and joined together with a silicone sealant to form a continuous vacuum tight assembly. The vacuum required for neutron economy is low (≲0.1 Pa); but, in order to prevent accumulation of material on the guide surfaces which would reduce the reflectivity, the actual vacuum is substantially better (≃1 mPa).

**Fig. 6:**
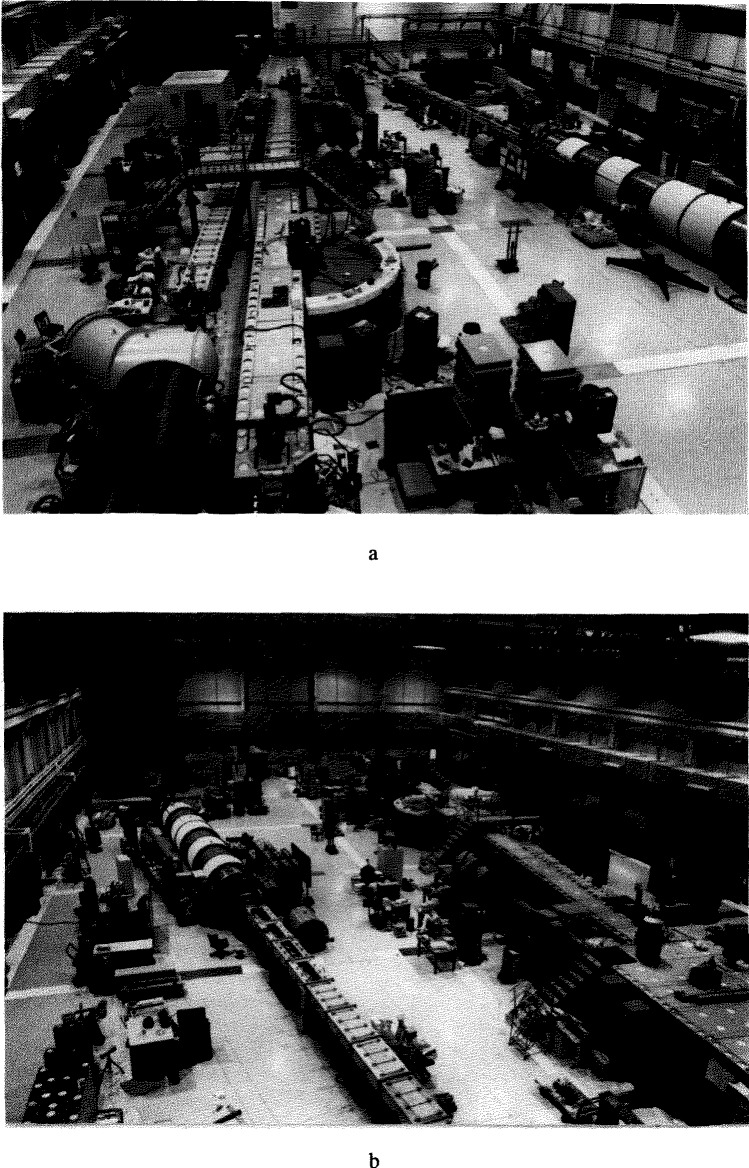
The guide hall (a) looking toward the confinement building; (b) looking away from the confinement building.

**Fig. 7:**
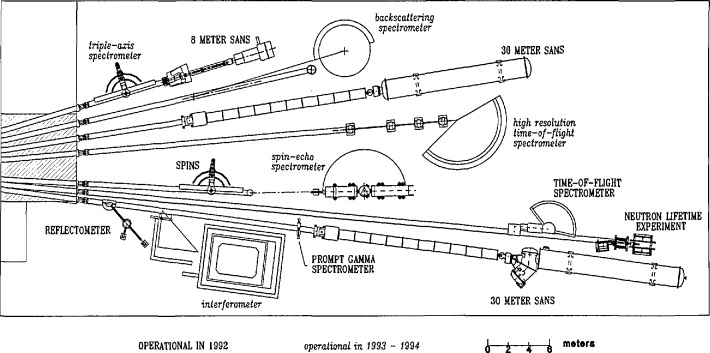
Floor plan of the CNRF on completion.

Each neutron guide serves several different experimental stations, and the system must allow all stations to operate simultaneously. Therefore, care must be taken in designing the methods used for beam extraction. This is accomplished in one of two different ways—either by dividing the height of the beams, so that a station uses a particular portion of the beam, or by inserting a device (usually a crystal monochromator) in the beam which removes a small fraction of the entire beam allowing the rest to continue for use further downstream. Experimental stations which must use the entire beam, or which must be directly in the beam line, are put at the end positions (which are in great demand). Examples of the latter type of instruments are the Small Angle Neutron Scattering (SANS) instruments, and the neutron lifetime experiment. Several of the instruments at various experimental stations are described in companion papers, which provide more details on the particular arrangements. Figure 6 shows two photographs taken in March 1992 showing the overall layout of the guide hall. Figure 7 shows a schematic drawing of the guide hall and associated instrumentation as it will exist when the facility is finished. As of March 1992, seven stations have been commissioned: an 8 m small-angle neutron scattering (SANS) spectrometer, a cold-neutron depth profiling facility, a neutron-optics test bench, a prompt gamma activation analysis station, a medium resolution time-of-flight spectrometer, a fundamental neutron physics station, and a high-resolution 30 m SANS spectrometer.

In addition to the guide hall, a new office/laboratory wing was built to support the hundreds of research participants that will utilize the CNRF for a broad range of different investigations. Space is provided therein for sample preparation, controlled temperature sample environmental assembly, and simple chemical preparation. A computer terminal room is also provided for simple data reduction and plotting, with terminals connected to a local area network that allows data transfer from the instruments to a local computer. From there, the data can be transferred to various media for transfer to the guest researchers’ home institutions for subsequent data analysis.

## 5. Neutron Instrumentation: An Overview

Neutron instrumentation at the CNRF encompasses a number of experimental techniques which include: those designed to determine the intrinsic properties of the neutron, neutron absorption for chemical analysis, and a range of neutron scattering instruments. In the case of neutron scattering it is appropriate to place in context the detailed discussions of instruments and applications that are described in the following articles.

Neutron scattering comprises a series of related techniques the object of which is to measure either of two cross sections (the details of which are elucidated in the following paper by N. F. Berk)
*dσ*(*Q*)/d*Ω*= the angle-dependent differential cross section integrated over energies.*d*^2^*σ*(*Q*,*ω*)/d*Ω*d*ω*= the energy- and angle-dependent double differential cross section.The power of neutron scattering techniques stems from the enormous range over which ***Q***, the wave-vector transfer, and ℏ*ω*, the energy transfer, can be varied, and the relevant cross sections evaluated. Of particular interest for our purposes here is the extension of range that cold neutrons provide.

**Fig. 8:**
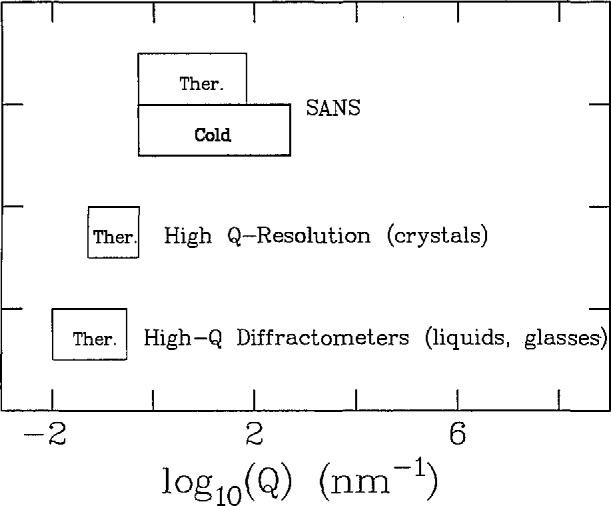
The wave-vector transfer ranges for neutron instruments which measure d*σ*/d*Ω*.

**Fig. 9:**
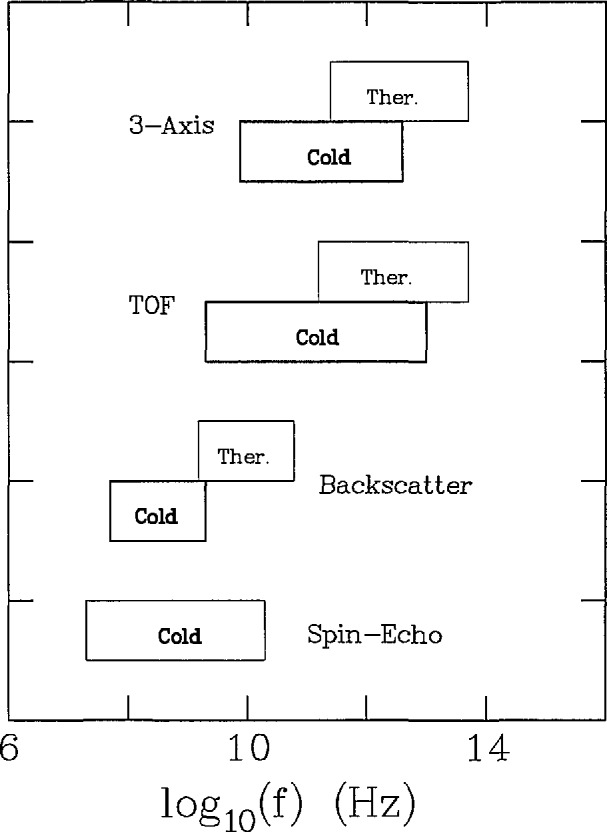
The accessible vibrational frequency ranges for neutron instruments which measure d^2^*σ*/d*ω*d*Ω*.

In Fig. 8 are shown the size regimes probed by the various techniques by which d*σ*-(***Q***)/d*Ω* is measured. It is clear that cold neutrons provide an extremely important enhancement of the SANS technique. This has been utilized for a number of materials science applications such as the evolution of pore size in ceramics and precipitates in metals. In Fig. 9 the techniques used to measure d^2^*σ(Q,ω)*/d*Ω*d*ω* are indicated according to the approximate range of frequency of motion which each probes. It is clear that for a given instrument type, the availability of cold neutrons extends the accessible range of dynamics to significantly lower frequencies.

In Table 1 some representative bound-atom scattering lengths and cross sections are given to illustrate the randomly varying sensitivity of neutrons to various elements. As described in detail in the following paper by N. F. Berk, the scattering length relates to the details of the neutron-nucleus interaction, whereas the cross section is a measure of the probability for scattering.

## 6. Outside Participation

It is planned that on completion the number of research stations associated with the CNRF will number 15 (capacity for two or three additional experimental stations will exist at the end of the funded project) and will be of two types. NIST will develop nine experimental stations for the use of the general United States science community. Two-thirds of the available time on these stations will be allocated by a Program Advisory Committee (PAC) on the basis of scientific merit of written proposals. The PAC has been appointed by NIST with a majority of members chosen from outside NIST. The other mode of operation will involve Participating Research Teams (PRTs), which develop and provide continuing support for the additional stations. These PRTs may include NIST although this is not necessary. Proposals from prospective PRTs are selected by the facility manager with the advice of the PAC.

The PRTs are responsible for the design, construction, maintenance, and up-grading of the facilities, in return for which they receive 3/4 of the available time. The remaining time is allocated by the PAC in the usual manner according to the proposal review system. For all instrumental stations, instrument-responsible scientists will be designated to assist users in the performance of their experiments. As of May 1992 PRT agreements include the following:

- Exxon Engineering and Research Corporation has participated in the design and construction of the CNRF’s first 30 m high-resolution small-angle neutron scattering (SANS) spectrometer, now operational. The University of Minnesota, through its Center for Interfacial Engineering (a collaboration among academia, government, and some 30 affiliate companies) is also a member of the PRT for this instrument.
- In a special form of PRT, NIST and the National Science Foundation (NSF) are developing a Center for High Resolution Neutron Scattering (CHRNS) for research in chemistry, physics, biology, and materials science at the CNRF. The center, comprising two state-of-the-art neutron research instruments being constructed by NIST with funding from NSF, is open to use by U.S. universities, industries, and government agencies. Approximately 75 percent of the NSF-funded portion of CHRNS will be scheduled entirely by the PAC, with about 25 percent of time set aside for instrument improvement, “breakthrough” experiments, and a small allotment of time for instrument-responsible scientists. The CHRNS instruments are the (second) 30 m SANS spectrometer and one-half of the spin-polarized inelastic neutron scattering (SPINS) spectrometer.
- A cold-neutron reflectometer is being developed by NIST in a PRT with IBM and the University of Minnesota.
- The Organic and Electronic Materials Department of Sandia National Laboratories (SNL) has also entered into a long-term cooperative research agreement with the CNRF. SNL will provide partial funding for development and support costs of the new ultra-high-resolution time-of-flight spectrometer and the new back-reflection spectrometer.
- Additional outside support and cooperation, already in place, includes a grant from Kodak for the chemical-analysis facilities, along with some equipment funding for Fundamental Physics applications by NSF—through the University of Missouri—and Department of Energy, and for cold-neutron crystal spectrometry by Johns Hopkins University. Further participation by outside groups is anticipated before the project is complete in 1993.

## 7. Outlook

In partnership with industry and government, NIST’s mission includes providing services based on science and technology to enhance the competitive posture of the United States in global markets. To underpin the technology programs, NIST has a broad range of scientific programs which are motivated by NIST’s charter to provide technical services to the scientific and engineering communities.

The CNRF is an outstanding example of the realization of NIST’s new mission in the context of its original charter. In addition to programs of fundamental scientific importance, it provides academic, industrial, and government scientists and engineers with advanced measurement technologies for research directly relevant to the materials, information and communication, chemical, electronics, and biotechnology industries.

Efforts to educate the scientific and engineering communities in the use of neutron techniques have met with dramatic success as evidenced by the increase in research participants over the last several years (even without the CNRF). Since the initiation of the CNRF a number of workshops have been held which have focussed on the broad range of cold neutron applications: from fundamental studies of the neutron, to macromolecules and microstructure studies, to cold neutron spectroscopy, to chemical analysis of advanced materials.

In the following papers some of the instruments of the CNRF are described in detail. Within each paper an attempt is made to illustrate the variety of applications possible for that instrument type, and insofar as possible, how CNRF capabilities compare with or extend the state-of-the-art of cold neutron research worldwide.

## Figures and Tables

**Table 1:** Neutron and x-ray scattering characteristics^a^,^b^,^c^

Element/isotope	*b*_coh_(fm)	*σ*_coh_(fm^2^)	*σ*_tot_(fm^2^)	*f*_x-ray_(fm)
^1^H	−3.74	1.76	81.7	0.2
^2^H	6.67	5.59	7.6	0.2
C	6.65	5.55	5.55	4.8
N	9.36	11.01	11.50	5.3
O	5.80	4.23	4.23	6.2
Al	3.45	1.50	1.50	15.5
Fe	9.54	11.44	11.83	33.0
W	4.77	2.86	4.86	114.0
U	8.42	8.90	8.91	148.0

a Neutron characteristics from V. F. Sears, in Methods of Experimental Physics: Neutron Scattering, Vol. 23A, eds. K. Sköld and D. L. Price, Academic Press, Inc. (1986) pp. 521–550.

b *b*_coh_= coherent scattering length, *σ*_coh_ = bound-atom coherent scattering cross section, *σ*_coh_ = coherent + incoherent scattering cross sections.

c Atomic scattering factors for x rays (sin *θ*/λ =5 nm^−1^) from G. E. Bacon, Neutron Diffraction, 3rd Edition, Clarendon Press, Oxford (1975) pp. 38–41.

